# Functional Changes of the Genitourinary and Gastrointestinal Systems before and after the Treatment of Endometrial Cancer—A Systematic Review

**DOI:** 10.3390/jcm10235579

**Published:** 2021-11-27

**Authors:** Marcin Oplawski, Agata Średnicka, Aleksandra Dutka, Sabina Tim, Agnieszka Mazur-Bialy

**Affiliations:** 1 Department of Gynecology and Obstetrics with Gynecologic Oncology, Ludwik Rydygier Memorial Specialized Hospital, Zlotej Jesieni 1, 31-826 Kraków, Poland; agata.srednicka@gmail.com (A.Ś.); olacora@gmail.com (A.D.); 2 Department of Gynecology and Obstretrics, Faculty of Medicine and Health Sciences, Andrzej Frycz Modrzewski University, Gustawa Herlinga-Grudzińskiego 1, 30-705 Kraków, Poland; 3 Department of Biomechanics and Kinesiology, Faculty of Health Science, Jagiellonian University Medical College, Skawińska 9, 31-066 Kraków, Poland; sabina.tim@doctoral.uj.edu.pl

**Keywords:** endometrial cancer, pelvic floor prolapse, POP, OAB, LUTS, overactive bladder, urinary incontinence

## Abstract

**Simple Summary:**

Endometrial cancer is currently one of the most common gynecological cancer and accounts for around 5% of all female cancers. The treatment strategy most often includes surgery and adjuvant radiation therapy. Thanks to the high effectiveness of used treatment methods, the patients can live longer lives. Unfortunately their quality of life can be negatively affected by side effects resulting from weakening pelvic floor such as urinary incontinence, pelvic organ prolapse and fecal incontinence. In our paper we analyzed the studies published between 2010 and 2020 that touch upon the prevalence and management of pelvic floor dysfunction in endometrial cancer patients. Our results show increase in the incidence of pelvic floor disorders after various forms of endometrial cancer treatment and the need for more good quality research in the subject to be able to provide patients with holistic care focused on minimizing treatment side effects and prioritizing their quality of life.

**Abstract:**

The incidence of endometrial cancer (EC), which coexists with such civilization diseases as diabetes, obesity or hypertension, is constantly increasing. Treatment includes surgery as well as brachytherapy, teletherapy, rarely chemotherapy or hormone therapy. Due to the good results of the treatment, the occurrence of side effects of therapy becomes a problem for the patients. One of the large groups of side effects includes the pelvic organ prolapse, urinary and fecal incontinence. The aim of this study was to present current knowledge on the occurrence of pelvic floor dysfunction in women treated for EC. A literature review was conducted in the PubMED and WoS databases, including articles on pelvic floor dysfunction in women with EC. PRISMA principles were followed in the research methodology. A total of 1361 publications were retrieved. Based on the inclusion and exclusion criteria, 24 papers were eligible for the review. Mostly retrospective studies based on different questionnaires were evaluated. No prospective studies were found in which, in addition to subjective assessment, clinical examination and objective assessment of urinary incontinence were used. Studies show a significant increase in the incidence of pelvic floor disorders, including urinary incontinence, after various forms of EC treatment. We believe that assessment of complications after endometrial cancer treatment is clinically relevant. The review emphasizes the importance of programming prospective studies to prevent and address these disorders at each stage of oncologic treatment.

## 1. Introduction

Cancers of the female reproductive organs including the mammary gland account for more than 40% of all female oncological problems [1,2]. One of the most common gynecologic cancer today is endometrial cancer (EC) [1,2]. In GLOBOCAN global statistical studies endometrial cancer is the sixth most common malignancy among women and accounts for 4.8% of all female cancers [3]. The incidence of endometrial cancer which coexists with such civilization diseases as diabetes, obesity or hypertension is steadily increasing [4,5]. This cancer affects mostly, postmenopausal women, 90% of cases occur in women over 50 years of age [4,5].

The treatment of endometrial cancer depends on the stage of the disease, the degree of tumor differentiation and the general condition of the patient [6]. When surgery is initially possible, the grade of the disease is determined on the basis of the FIGO 2009 surgical-pathological classification with modifications, and in cases when surgery is impossible on the basis of the FIGO 1971 classification [7].

The surgical procedure depends on FIGO stage of the disease. Simple or radical hysterectomy with adnexectomy with or without pelvic and paraaortic lymphadenectomy is performed [6]. Complementary treatment of locally advanced tumors consists of brachytherapy and/or teletherapy. In cases of more advanced and generalized lesions, the treatment is additionally supplemented with chemotherapy and nowadays less frequently with hormone therapy [6]. The results of the treatment of this cancer are considered satisfactory, which is reflected in a five-year survival rate of more than 80% of patients [8].

Due to the good results of treatment [8], the occurrence of side effects of therapy often becomes a primary concern in these patients [9]. The consequences of treatment observed in a large group of patients include pelvic floor dysfunctions such as pelvic organ prolapse and urinary and fecal incontinence [10].

In a multicenter Polish study, it was found that as many as 10% of patients reporting pelvic organ prolapse had a previous abdominal hysterectomy [11]. During hysterectomy for oncological reasons, the vaginal stump is not fixed and thus the structures supporting the reproductive organ are not reconstructed, which increases the risk of subsequent disorders in pelvic floor statics. The literature also emphasizes the impact of intraoperative nerve damage that may lead to subsequent bladder dysfunction [10,12]. The most commonly used complementary treatment (i.e., radiotherapy) may contribute to pelvic floor lesions in the mechanism of radiation toxicity [13,14,15].

The problem of pelvic floor dysfunction after gynecologic cancer treatment is often treated perfunctorily in the literature, as it is in clinical management the oncology treatment-oriented cancer patient. However, dysfunction in the pelvic floor after cancer treatment is one of the main reasons for reduced quality of life in female patients [12], thus deserves attention. The quality of life is worsened by pelvic floor dysfunctions such as dysuria, overactive bladder, stress urinary incontinence, mixed forms of incontinence, urinary retention or urge incontinence, descent or prolapse of the reproductive organ, fecal incontinence [13,16].

In many centers treating EC, research is focused on finding equally effective but less invasive therapies and on preventing and treating the side effects of combined therapy. In surgical treatment, less invasive techniques such as laparoscopy or robotic surgery are being introduced, however no significant advantage of any of those surgical methods in the treatment of EC is observed [17,18]. In radiotherapy, brachytherapy and teletherapy doses are limited in combined treatment. Multicenter studies like PORTEC-3 [9] evaluate the efficacy of different models of combination therapy. The side effects of the treatment are evaluated by broadly assessing the quality of life of these women. Decreased quality of life in these patients is related to both the psychological and organic spheres. Gastrointestinal, genitourinary and nervous system disorders are the ones most commonly affecting patients’ quality of life [19].

The purpose of this review is to provide an overview of the current knowledge of pelvic floor dysfunction in women treated for endometrial cancer. We want to investigate the prevalence of pelvic floor disorders in women before and after oncological treatment, characterize acute and late bladder and bowel toxicities among EC patients treated with radiotherapy and describe existing research on therapy of pelvic floor disorders associated with EC treatment.

We believe that creating such a review will systematize the knowledge of this issue, will be important for making correct clinical decisions during endometrial cancer treatment, and will indicate the way for future research on pelvic floor dysfunction in patients with EC. In this paper we focus on one of the most important aspects affecting the comfort of life after endometrial cancer treatment (i.e., various forms of pelvic floor dysfunction with particular emphasis on urinary incontinence). In the case of these disorders, it is possible to introduce appropriate management before cancer therapy which may have a beneficial effect in reducing the incidence of complications in these women. It is also possible to apply physiotherapeutic and urogynecological techniques after the cancer treatment, including conservative as well as surgical treatment.

For this review, we qualified papers from 2010–2020, in English, that considered the occurrence of pelvic floor dysfunction at diagnosis and each stage of endometrial cancer treatment.

## 2. Materials and Methods

The review protocol was based on the Preferred Reporting Items for Systemic Review and Meta-Analysis (PRISMA).

The inclusion criteria were based on the Participant-Intervention-Comparator-Outcomes-Study design (PICOS) format.

*Participants*: Only papers which included women with endometrial cancer were qualified for the study. Publications on functional disorders of the pelvic floor in patients with cancer of the reproductive organs, which did not distinguish the group of patients with endometrial cancer, and publications that did not specify used treatment were excluded. The review was conducted by two independent authors.

*Intervention*: Study that contained any therapeutic intervention were included; study where it was possible to separate the results of intervention, if combined.

*Comparison*: No intervention, comparison with another method/intervention.

*Outcomes*: Pelvic floor dysfunction in endometrial cancer the effect of endometrial cancer therapy on the type, frequency and severity of pelvic floor dysfunction, symptoms of urinary incontinence, quality of life, urodynamic parameters.

*Study design*: Randomized controlled trials, publications in English. Non-experimental studies, reviews were excluded.

The literature review was conducted in PubMED and Web of Science databases, moreover Cochrane Central Register of Controlled Trials, and Scopus were searched. Inclusion and exclusion criteria were established for analysis of titles, abstracts, and full publications. Papers on the occurrence of pelvic floor dysfunction in women with endometrial cancer were included in the review. Articles in English only, published between 2010 and 2020, were eligible for inclusion. Exclusion criteria were as follows: language of the paper other than English, year of publication before 2010, no full-text version of the paper available. Systematic reviews, letters to the editor, master’s or doctoral theses, abstracts of conference presentations, and research protocols were also not included in the review. After searching for each phrase, the results were exported to an Excel sheet. Duplicates were removed after an overall search.

The search strategy and keywords were set as follows:

(endometrial cancer OR uterus cancer OR uterine malignancy)

AND

(uterine prolapse OR pelvic floor prolapse OR OAB OR incontinence OR LUTS OR urinary tract disfunction OR fecal incontinence OR overactive bladder OR neurogenic bladder OR overflow incontinence OR bladder disfunction OR rectocele OR cystocele OR enterocele OR urethrocele OR uterine prolapse OR pelvic floor symptoms OR toxicity)

The search was carried out by two researchers independently. The first screening of articles was evaluated by titles and abstracts against inclusion and exclusion criteria. After screening, all full text of included articles were retrieved. Any disagreements between the researchers will be resolved through discussion with another, third author.

The risk-of-bias analysis was performed by two researchers independently using Risk-of-Bias 2 tool, which is available on the Cochrane platform. Five domains of bias were evaluated in the analysis: randomization process; deviations from intended interventions; missing outcome data; measurement of outcome; and selection of the reported result. Each domain consisted of 3–7 questions, which could be answered as Yes/Probably Yes/Probably No/No/No information. Based on the answers provided, the tool assessed the risk of bias of each domain and of the entire study.

ROBINS-I analysis was carried out to assess the risk of bias of nonrandomized studies or interventions. Six domains of bias were evaluated in the analysis: confounding, participants, interventions; deviations from intended interventions; missing data; measurement of outcome; and reported results.

## 3. Results

**Figure 1:**
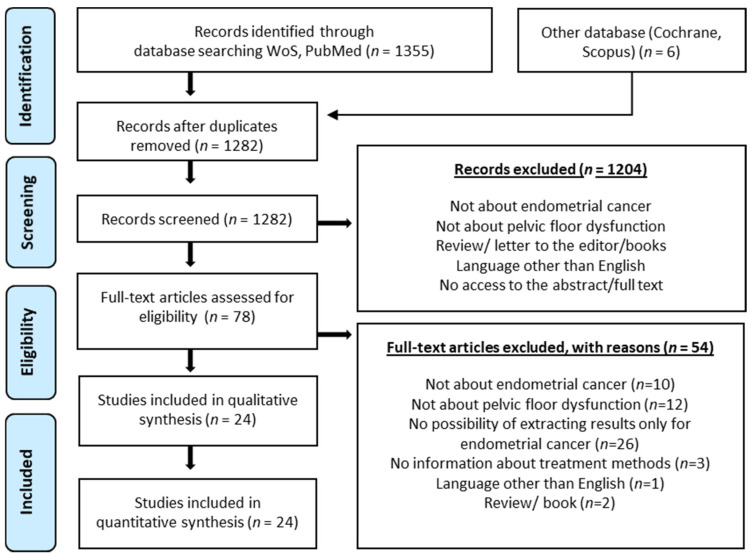
PRISMA diagram presenting the different phases of the systematic review.

**Figure 2:**
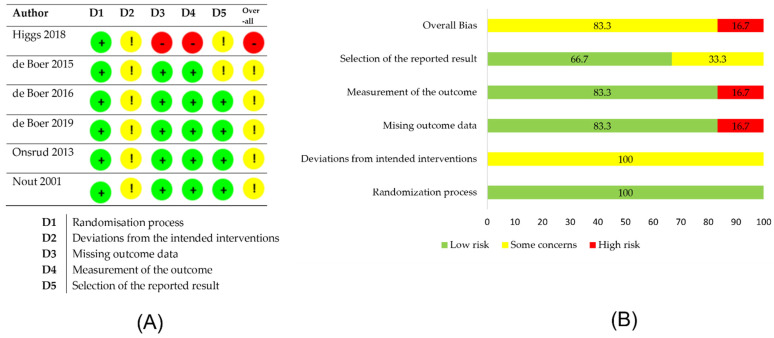
Risk-of-bias analysis (ROB-2) and summary plot of the overall risk of bias. (**A**) RoB-2 analysis. (**B**) Summary plot of the overall risk of bias.

A total of 1355 publications were searched in PubMED and Web of Science databases. Six publications found in other databases were added. After removal of duplicates, 1282 publications remained. Based on the inclusion and exclusion criteria, 24 papers were qualified for the review. The PRISMA diagram (Figure 1) was used to describe the different stages of the review. The diagram shows the reasons for publication exclusions and the final number of papers included in the analysis. The risk-of-bias assessment showed that the quality of included studies is moderate or questionable (Figure 2 and Table 1). Table 2 presents a brief description of the eligible studies.

### 3.1. Evaluation of Pelvic Floor Disorders in Women before Oncological Treatment

Patients diagnosed with gynecologic cancers often already have preexisting lower genital and urinary tract problems. This is related to the phenotype of these women, who are often over the age of 50, have a BMI over 25, diabetes and hypertension [36].

Bretschneider et al., 2016 [19] conducted a survey of women, prior to scheduled cancer treatment, on pelvic floor and urinary tract disorders using the RSC (Rotterdam Symptom Checklist) and ICIQ-FLUTS (International Consultation on Incontinence Questionnaire-Female Lower Urinary Tract Symptoms) questionnaires. The evaluation involved 152 women with genital cancers, which included endometrial cancer, ovarian cancer, and vulvar cancer. More than half of these women (59.5%) reported symptoms of urinary incontinence (UI), including more than one-third (33.9%) of urge urinary incontinence (UUI). There were no statistically significant differences between cancer types. It was noted that in older women (over 50 years of age) urinary urgency was more frequent than in younger patients. Patients with endometrial cancer constituted 61.8% of the study group. Among them, 37% reported symptoms of urinary incontinence. The detailed characteristics of the abnormalities in patients with EC are presented in Table 2. The disadvantage of this study was that it was based on questionnaires only, without a clinical examination, and significant differences were found in the results depending on the questionnaire used.

The similar results were presented by Thomas et al. [17] who evaluated based on their own questionnaire 549 women presenting to a gynecologic oncology clinic with suspected cancer, 347 of whom were diagnosed with genital cancer. Among patients with EC, urinary incontinence was found in 55%, with stress incontinence in 36% and urgency in 14.5%. In addition, in this study, there was no statistically significant difference in the incidence of UI between gynecologic cancer types. There was also no statistically significant difference in the incidence of UI between groups of patients with neoplastic lesions and benign lesions (*p* = 0.89).

The above studies have also highlighted other pelvic floor disorders occurring in patients with EC before oncological treatment. Bretschneider at al. [19] found fecal incontinence in 3% of patients with EC. This is the least common disorder reported in this study, but in a large proportion of cases (approximately 50%), patients report a negative impact of bowel symptoms including FI on their quality of life.

In a study by Thomas et al. [17], questionnaires assessing genital prolapse were used. POP was found in 7% of patients, and also POP was not described to be more frequent in cancer patients than in controls (*p* = 0.2). The data presented in this study show that 20% of patients consider POP or UI to be symptoms that moderately or severely impair their quality of life.

In the general female population, urinary incontinence problems affect approximately 30% (5–70%) [35], whereas 51–59% in patients before oncological treatment [17,19].

The results of the above studies do not clearly show a higher rate of incontinence problems and other pelvic floor disorders (POP, FI) in patients before gynecological cancer treatment than in the general population. The authors of papers suggest that the occurrence of urinary incontinence may be correlated with the age or BMI of patients rather than associated with a specific type of cancer, but there is no evidence to support this. The present study focused on showing differences in the incidence of particular pelvic floor disorders between patients with different gynecological cancers, but did not relate the results to the general population, which may point to the direction of further research needed.

It should be mentioned that these studies were based only on subjective questionnaire assessment without being complemented by objective techniques such as clinical or urodynamic examination, which may indicate their weakness. The detailed characteristics of the studies are summarized in Table 3.

### 3.2. Evaluation of Pelvic Floor Disorders in Women after Surgical Treatment of Endometrial Cancer

The proximity of a female reproductive system with other organs within the pelvis determines the possibility of interaction between them during surgical treatment. As mentioned before simple hysterectomy with adnexal removal with no vaginal cuff or parametria is performed in FIGO stage I–II EC and radical hysterectomy is performed in patients with higher FIGO stages of the disease. Lymphadenectomy is performed in FIGO stage II- IIIB, whilst in FIGO stage I it can be performed based on individual risk assessment. The effect of uterine removal on pelvic floor function problems is described in the study by Nosti et al. [21], examining 25 women at a minimum of six months after traditional transabdominal surgery using the Pelvic Floor Distress Inventory (PFDI-20) and Urinary Distress Inventory (UDI-6) questionnaires. Pelvic floor disorders were found at a much higher frequency (84%) than in the general population. Urinary incontinence problems of varying severity occurred in 76%, symptoms related to lower gastrointestinal disorders occurred in 68%, and symptoms of pelvic organ prolapse were observed in 44% of the women studied. Urinary tract-related symptoms had the greatest impact on patients’ quality of life. While this study showed a high prevalence of pelvic floor disorders in women after abdominal hysterectomy for endometrial cancer, it was performed on a small number of women and did not include a preoperative urogynecological assessment. However, it is consistent with previous works on the effect of uterine removal on urinary continence problems, observed in approximately 80% of patients after endometrial cancer treatment [37].

To reduce the risk of complications, procedures to protect the vegetative innervation (NSRH) [38] and minimally invasive techniques such as laparoscopy (TLH) and robotic surgery are being introduced [20,39,40].

A comparison of pelvic floor disorders after laparoscopic removal of the uterus with lymph nodes with the traditional method was presented by Higgs et al. [20]. In this multicenter prospective randomized phase III LACE study, 381 patients were evaluated with the PFDI-20 questionnaire before surgery and at six-month intervals after TLH (186 patients) and TAH (195 patients) for early stage endometrial cancer. There was an initial improvement in pelvic floor function in both treatment groups, mainly in terms of pelvic organ prolapse and urinary problems. Women in the TLH group had a lower pelvic floor function score on the PFDI-20 scale, but the difference between TAH and TLH was not statistically significant. There was no difference in pelvic floor symptoms in patients who received adjunctive treatment compared to those treated with surgery alone. The study indicates that genitourinary symptoms are not likely to deteriorate after abdominal or laparoscopic hysterectomy. In contrast, Lipetskaia et al. [18] compared the effect of robotic surgery with and without lymphadenectomy in endometrial cancer treatment on urinary problems in a retrospective cohort study of 74 women. The study used the UDI-6 and IIQ7 (Incontinence Impact Questionnaire) questionnaire to assess urinary dysfunction. The main focus was on symptom assessment between patients with and without lymphadenectomy (control group) with no statistically significant differences between the groups. However, the percentage of urinary incontinence increased significantly in the study population, from 28% before surgery to 74.3% after the robotic procedure. The study did not evaluate other pelvic floor disorders.

The change in surgical method was presented from another point of view in the article by Paek et al. [41], where the techniques of protecting the vegetative innervation during surgery (nerve-sparing radical hysterectomy; NSRH) due to gynecological malignancies, including endometrial cancer and its effect on urinary dysfunction in treated women were indicated. In this study, rapid recovery of bladder function was observed after NSRH. This indicates a direction for change in the surgical management of endometrial cancer to minimize damage to the innervation of the pelvic organs.

In conclusion of the above mentioned studies surgical access and the extent of the procedure in terms of lymph node removal are not significant in the appearance of incontinence problems. The detailed descriptions of the studies are presented in Table 4.

### 3.3. Evaluation of Pelvic Floor Disorders in Women after Radiotherapy in Endometrial Cancer Treatment

In the case of endometrial cancer, the most common treatment complementary to surgery is radiation therapy consisting of brachytherapy (VBT) and/or teletherapy (ERBT), depending mainly on the stage of the disease.

A retrospective survey by Segal et al. [22] evaluated 149 women with EC, of whom 41% received complementary radiotherapy (brachytherapy 28 women VBT, whole pelvis teletherapy 34 women ERBT). Urinary incontinence was found in 53% of patients in the entire study group. The percentage of post-radiotherapy patients who reported urinary incontinence was 57.5%, among them 21% reported stress urinary incontinence and 13% had overactive bladder. The results showed no statistically significant differences between the complaints of patients who did not undergo radiotherapy (NAT) and those who received it, *p* = 0.47. The conclusion of the study was that radiotherapy does not affect urinary symptoms as much as age and obesity. Some shortcomings of this study are the lack of evaluation of patients before EC treatment and the placement of brachytherapy and teletherapy patients in one group. These groups were separated and such an assessment was performed in the PORTEC-2 study by de Boer et al. [14] focusing on various aspects of quality of life after EC treatment including incontinence. In the study, 427 patients were randomly assigned to either VBT or EBRT as follow-up treatment. The study reported significantly more frequent moderate-to-severe UUI in patients after ERBT than VBT after seven years of follow-up (39.3 vs. 25.5%, *p* = 0.05), while no difference was observed for SUI (50.6% vs. 50.9%). In contrast, the study by Oplawski et al. [23] compared a group of women after uterine removal for non-oncological reasons (23 women) with a group of women after surgical treatment supplemented with VBT (23 women). The study found a significant negative effect of VBT on urinary tract disorders at six and 12 months after the treatment. Undoubtedly, hysterectomy is a risk factor for disorders of pelvic statics and urinary incontinence therefore, in most cases of uterine removal for non-oncological reasons, suspension of the cervix/vaginal vault to the obtuse ligaments, sacro-uterine ligament or Cooper’s ligament is performed (sp. Burch surgery) [42]. The aim is to prevent the occurrence of statics disorders in the future. The standard surgical treatment in EC is modified radical hysterectomy without vaginal vault suspension. Therefore, the results of this study may be influenced by the fact that researchers compared women with uterine removal without fixation (oncologic procedures) to those in whom the cervix was preserved and/or the suspensory apparatus was reconstructed.

Another reason for the increased incidence of UI after VBT may be the adverse effects of radiation on the vaginal mucosa, as shown by Bahng et al. [24] in a study evaluating 100 women after complementary brachytherapy treated due to EC. This study showed that vaginal mucosal dysfunction affects up to 47% of patients after VBT, and in 14% of them it causes complaints that can be classified as grade 2 or 3 according to the Common Toxicity Criteria for Adverse Events v. 4.02. This means that these patients have subjective discomfort related to vaginal narrowing, shortening and dryness, sexual dysfunction and abnormalities on gynecological examination. In contrast, an evaluation of the effect of ERBT after surgery was presented by Nout et al. [13] in the PORTEC-1 study, where a statistically significant negative effect of ERBT on urinary problems was found compared to no follow-up treatment (NAT). The study had a follow up of seven years and involved 246 women with EC, 113 of whom received EBRT radiotherapy. Urinary incontinence was found in 57.8% of patients after radiotherapy and 38.2% of patients in the NAT group, respectively. The need for the use of pads and other hygiene products due to incontinence was reported by 42.9% of patients in the EBRT group and only 15.2% of patients in the non-radiotherapy group. NAT patients with regional tumor recurrence had a higher incidence of UUI and SUI comparable to the EBRT group (UUI *p* = 0.078, SUI *p* = 0.090). The conclusion of the PORTEC-1 study was that due to urinary and gastrointestinal disorders, among others, ERBT should be avoided in stage I low- and intermediate-grade EC.

A small group of patients, for various reasons, are treated only with radiotherapy. Such treatment approach is shown in a study by Kauffmann et al. [25] on a group of six women, mostly with comorbidities, treated only with brachytherapy (VBT—HDR) or stereotactic teletherapy (ERBT—SRBT). In both groups, the doses were much higher than those given as standard, due to the tumor stage or constitutional conditions of these women. The results showed a higher incidence of urinary tract abnormalities in patients undergoing ERBT than in patients after VBT-HDR. Early abnormalities occurring during radiotherapy were evaluated. In conclusion authors indicated VBT as the gold standard of EC treatment in non-operative cases. In this study, urinary tract abnormalities were presented collectively without distinguishing SUI and UUI. The disadvantage of this study is a very small study group.

Evaluation of, among others, urinary tract disorders was presented in an extensive cohort study by Soisson et al. [16] evaluating the long-term effects of successful treatment of EC. Patients were compared to a population without endometrial cancer. The study included 2648 patients with EC, 68.5% of the women had a surgical procedure only, 21.9% had surgery and radiotherapy without specifying the type, 3.2% had surgery and chemotherapy, and 4.7% had surgery and radiochemotherapy. Follow-up lasted five and 10 years after the treatment. The relative risk of urinary tract disorders was determined in the group of patients who underwent surgery and radiotherapy compared to patients who underwent surgery only, finding a higher risk in the group undergoing RTH one to five years after the treatment but not >five years after the treatment (HR up to five years—1.46, five to 10 years—1.24). It is worth noting that the urinary disorders described here do not include UI but rather involve conditions such as urinary tract infections, kidney stones, renal failure, etc. The authors suggest that UI may occur in patients with EC secondary to the urinary tract disorders studied.

Emidar et al. [26] used urodynamic evaluation in their study but this work compares three small groups of women treated for non-advanced cervical cancer (Gr1), endometrial cancer (Gr2) and advanced cervical cancer (Gr3). Piver III radical uterine removal with radiotherapy (Gr1), Piver II radical uterine removal with lymph node removal and radiotherapy (Gr2) or radiotherapy only (Gr3) were performed. After the treatment, compared with the urodynamic study performed before treatment, no significant differences were observed in any of the three groups in terms of first urinary urge and urinary retention. In addition, no post-treatment changes in maximum bladder pressure (MVP) or maximum detrusor pressure (MDP) were observed in the EC group, but there was a statistically significant increase in urinary incontinence, decrease in normal (NUUV) and strong (SUUV) urge, decrease in bladder capacity and urethral mobility. The results suggest complementary radiotherapy following radical hysterectomy may result in lower urinary tract dysfunction.

Urgency, frequent urination and incontinence are a group of disorders occurring after combined treatment of endometrial cancer (surgery + RTH) described in many publications. However, the data presented in the cited studies show that radiotherapy also has effects on other pelvic floor functions. In a study by de Boer at al. [14], fecal incontinence and bowel disorders such as diarrhea were found to be more frequent in patients undergoing EBRT compared to the group treated with VBT. The significant impact of these complaints on the patients’ quality of life at 10 years post-treatment was highlighted. Similar results were presented in the study by Nout et al. [13] where frequent bowel movements, fecal incontinence and diarrhea were statistically more frequent in patients undergoing EBRT compared to the group not treated with radiotherapy. Similarly, statistically significant limitation of daily functioning due to these complaints was found. The detailed characteristics of the study are summarized in Table 5.

The study by Kauffmann et al. [25] found a higher incidence of bowel complications (according to CTCAE v.4 criteria) in patients undergoing ERBT than in patients after VBT-HDR. In contrast, Segal et al. [22] found no increased risk of fecal incontinence in patients after RTH, but described a significantly increased risk of sexual dysfunction. In this study, there is very little data on pelvic organ prolapse after the treatment. Segal et al. [22] based on a questionnaire survey found POP in 3.4% of patients before the treatment and 6.5% after combined treatment, the difference in occurrence was not statistically significant. This is lower than the incidence of POP in the general population reported in the literature [43,44,45,46]. Inconsistency may be due to the questionnaire nature of the study without having the patients evaluated by a clinician, which significantly decreases the reliability of the result. Numerous organ toxicity scales, such as RTOC, EORTC, CTCAE, have been developed to help assess the severity of complications following radiotherapy. They classify specific symptoms from different organs on a three-grade scale, where 1 corresponds to mild/mildly severe symptoms and 3 to severe symptoms, considerably worsening the patients’ quality of life. In relation to EC, scales describing bowel and bladder complications are most commonly used—these are the pelvic organs with the highest radiation exposure during radiotherapy.

Kuku et al. [27] described a group of 73 patients after combined endometrial cancer treatment who reported gastrointestinal disturbances persisting for more than three months during follow-up. The treatment included teletherapy (EBRT). It was found that, on average, symptoms appeared eight months after the end of radiotherapy, and in 37.3% of patients they significantly affected the quality of life. The study characterized 14 typical symptoms among which the most common were urgency, frequent defecations, abdominal pain, diarrhea, bloating, fecal incontinence and rectal bleeding. At the time of the last follow-up 10 years after radiotherapy, 28% of the patients had their bowel symptoms resolved completely, 51% had mild symptoms controlled with diet and loperamide and 2.6% of the patients experienced severe symptoms. Conclusions are optimistic, as they indicate resolution of severe bowel symptoms over time, and easy management of symptoms that persist chronically.

Vandecasteele et al. [28] characterized the incidence of early and late toxicities involving the gastrointestinal and urinary tracts after the modern technique of IMRT teletherapy. Modulation of beam intensity (IMRT) allows for dose differentiation in the irradiated area and a reduction in the dose received by healthy tissues, thus likely leading to fewer complications [47]. A total of 41 patients with endometrial cancer while receiving IMRT radiotherapy were evaluated in terms of toxicity. Acute gastrointestinal toxicity was reported in 93%, of which most patients had grade I toxicity. The most common problems reported were frequent defecations, abdominal pain, and urgency. Fecal incontinence occurred in 7% of patients. Acute urinary tract complications concerned 65% of patients, also in this case the first grade of symptoms predominated, the most frequent of which were: nycturia, urgency, pollakiuria. The evaluation of late complications was performed in the group of 25 patients. The incidence of complications from both systems was the same and amounted to 36%. The majority of complaints were grade 1. Among late complications, incontinence was the most common with 20% of patients. The detailed data are summarized in Table 6.

Early complications after IMRT radiotherapy were also described by Barillot et al. [29]. Early toxicity of at least grade 2 involving the gastrointestinal and urinary tract has been shown to be less than 30% with this technique. Any gastrointestinal side effects were found in 85%, most grade 1. The most common symptoms included diarrhea, inflammatory bowel disease and occasionally fecal incontinence. The authors suggest that the additional use of BRT did not increase the incidence of toxicity symptoms. Any symptoms of urinary toxicity were described in 39.5% of patients, most grade 1, with cystitis and pollakiuria predominating. Most symptoms occurred between three and five weeks after the start of treatment. On gynecologic examination, most patients showed redness of the vulva and vagina (vaginal erythema, grade 1).

The study by Roszak et al. [30] described early and late toxicities after EBRT radiotherapy with subsequent brachytherapy. Higher rates of early and late gastrointestinal than urinary tract complications were observed (*p* < 0.004). Early gastrointestinal toxicity occurred in 26.5% of patients, while bladder toxicity occurred in 18.5% of patients. Late gastrointestinal toxicity occurred in 7.4% of patients and bladder toxicity occurred in 1.5% of patients. Mild toxicity significantly predominated in all groups. The study found that older patients had a higher risk of complications.

Samper-Ternent et al. [31] compared the incidence of early and late toxicity in patients treated with and without radiotherapy. The limitation of the study is the lack of consideration of the type of radiotherapy received, while the advantage of the study is the large group of patients. Early gastrointestinal toxicity occurred in 21.9% of patients, late in 60.8%. Each type of toxicity was significantly more frequent in patients undergoing RTH, the most common being inflammation and bleeding. Early bladder toxicity occurred in 14.3% of patients, late in 35.8%. There was no statistically significant difference in the occurrence of early toxicity between the groups. Most patients reported urinary incontinence and bleeding. Most toxicity symptoms were mild to moderate; only 10% of women after RTH required readmission for gastrointestinal complications; less than 1% of women after RTH required admission for urinary complications. Risk factors for late complications included older age, history of chemotherapy, comorbidities, gastrointestinal and urinary tract symptoms before diagnosis, and history of early complications from radiotherapy.

The study by Onsrud et al. [32] evaluated the long-term outcomes of endometrial cancer (grade 1) treatment. They compared a group of patients undergoing adjuvant brachytherapy with a group undergoing combined adjuvant EBRT + VBT treatment. The main aim of this study was to evaluate the prognosis after both types of radiotherapy—there were no statistically significant differences in survival time from the beginning of treatment between the groups, however, a significantly higher incidence of grade 2 toxicity assessed by the French-Italian Glossary scale was observed in patients after EBRT (27.4% vs. 4.5%). A limitation of this study is the evaluation of toxicity after radiotherapy given more than 20 years ago and, therefore, with different technology and doses than used today.

Radiation therapy is a very important part of endometrial cancer treatment. It is most often used as an adjunctive treatment and only in inoperable cases alone. Two types of radiotherapy are used (i.e., brachytherapy and teletherapy). Brachytherapy is much safer for patients in terms of subsequent functional disorders within the pelvis, including urinary incontinence. It was shown in the multicenter PORTEC study over several years that the gold standard treatment is surgery and brachytherapy. Unfortunately, despite the use of increasingly newer technologies, the impact of radiotherapy on the urinary and gastrointestinal systems is relatively high, especially as an early radiation reaction (up to six months after the treatment). There is a lack of consistency in the incidence of toxicity, but the authors agree that early and late complications of EC radiotherapy more often affect the gastrointestinal tract than the urinary system. Most complaints are mild and transient, are amenable to conservative therapy, and rarely require re-hospitalizations. Most studies describe a decrease in the incidence and severity of toxicity over time. There is a lower rate of complications after brachytherapy and IMRT teletherapy compared to conventional radiotherapy, which is reflected in the current standard of care.

### 3.4. Evaluation of Pelvic Floor Dysfunction in Women after Chemoradiotherapy in the Treatment of Endometrial Cancer

In more advanced cases of endometrial cancer, radiochemotherapy is used after surgery, while in primary disseminated cancer chemotherapy is used [47]. The mortality rate in these cases is very high and consequently there is little work on pelvic floor disorders. In the previously cited study by Soisson et al. [16] evaluating the distant outcomes of endometrial cancer treatment, 84 (3.2%) women had surgery and chemotherapy and 124 (4.7%) had surgery and radiochemotherapy. Follow up at one to five years and five to 10 years after treatment. The group in which surgery was performed was compared in terms of urinary tract problems with patients after adjuvant radiotherapy (HR one to five years—1.46, HR five to 10 years—1.24), and chemotherapy (HR one to five years—2.99, HR five to 10 years—0.74), radiochemotherapy (HR one to five years—2.34, HR five to 10 years—1.51). The results suggest negative impact of adjuvant chemotherapy on urinary tract mainly in the early period after the treatment. It is noteworthy that urinary system disorders described in the study do not include urinary incontinence, but problems like urinary tract infections, nephritis, renal failure, etc. Authors hypothesize that urinary incontinence might be secondary to above mentioned urinary diseases. The results of this study indicate that on the one hand complementary treatment has a beneficial effect on the treatment of the underlying disease, but is associated with an increased risk of side effects, here urinary tract disorders. It should be noted that this was a very large population study, but only a survey.

In the multicenter randomized PORTEC-3 trial, quality of life was assessed as one of the outcome measures. The study by de Boer et al. [33] compared 333 women undergoing complementary radiotherapy and 327 radiochemotherapy. The critical time point of 24 months post-treatment was reached in 170 and 194 women, respectively. Patients in the chemoradiotherapy group received two cycles of cisplatin 50 mg/m^2^ during the first and fourth weeks of radiotherapy, followed by four cycles of carboplatin under the curve (AUC) 5 and paclitaxel 175 mg/m^2^ at 21-day intervals (and a 28-day interval between the second concurrent and first adjuvant cycles). EORTC QLQ-C30 (European Organization for Research and Treatment of Cancer Quality of Life Questionnaire Core 30) questionnaire was used indicating statistically lower quality of life in patients who received radiochemotherapy; in case of urinary tract disorders, there were no statistically significant differences, obtaining 17% after radiotherapy and 21% of SUI and UUI after chemoradiotherapy, respectively. The PORTEC series of studies is dedicated to the treatment of EC with the aim of maximizing treatment outcomes and assessing the side effects of radiotherapy, the evaluation of urinary tract was one of the components of the original quality of life questionnaire. The studies shown do not suggest that current chemotherapy regimens have an impact on urinary incontinence in women.

The study by de Boer et al., 2019 [20] is a follow-up of the PORTEC 3 series after five years. The final analysis included 201 patients undergoing CHRTH and 187 patients treated with RTH. Adjuvant chemotherapy for high-risk endometrial cancer was shown to improve the five-year recurrence-free survival of patients, but there was no statistically significant improvement in five-year overall survival. There was no statistically significant difference in the incidence of urinary incontinence disorders. There was no statistically significant difference in the incidence of grade 2 and 3 genitourinary and gastrointestinal adverse events. Details are shown in Table 7. To sum up, there is no clear evidence from RCTs that adjuvant chemotherapy is increasing risk of urinary incontinence in endometrial cancer patients, at the same time it has been shown that it is increasing risk of gastrointestinal adverse events during treatment that tend to cease quickly. Those conclusions are based on papers by De Boer [9,33], both of which refer to the same population selected for the PORTEC-3 randomized trial. The small number of available papers on the subject meeting our inclusion criteria indicates the need for further research.

### 3.5. Management of Urogynecologic Disorders during Cancer Treatment in Women

The treatment of reproductive organ statics abnormalities during oncologic treatment is not popular. An assessment of this issue is presented in the work of Bochenska et al. [35], who studied 23,501 women treated for gynecologic cancers between 2010 and 2014. Data were obtained from available surgical registries of the American College of Surgeons National Surgical Quality Improvement Program. 63% of these women were treated surgically for endometrial cancer; only 2.4% had concomitant POP repair surgery. The authors did not evaluate the effectiveness of reconstructive surgery, they focused mainly on 30-day postoperative adverse events assessment. They conclude that this type of surgery is too infrequent during the primary operation, while the use of these techniques could improve women’s quality of life after EC treatment without increasing surgical risk.

Similar findings regarding the frequency of performing the concurrent urogynecological surgery were published by Bretschneider et al., 2018 [15] who evaluated using the same database 25,138 women treated surgically for gynecologic malignancies between 2013 and 2016. Authors assessed postoperative complications in both groups concluding that combined reconstructive and oncologic surgery is not associated with significant increase of postoperative adverse event risk. However, they found out slightly higher rate of blood transfusions in combined surgery group (7.2% vs. 3.6%, OR 1.7, 95% CI 1.1–2.8). Both of the above mentioned studies are based on the data obtained from the same registry from partially overlapping years. A pilot study by Robinson et al. [34] presented data on the concurrent treatment of patients with endometrial cancer and additionally reporting SUI. The original study enrolled 55 women with endometrial cancer of whom urinary incontinence was reported in 23 (39%) women. In 15 women, stress urinary incontinence was treated surgically in eight cases, conservatively in two, and only observation was used in five cases. The study included too few patients to show a statistically significant difference. The study demonstrates that coexistence of urogynecologic disorders and endometrial cancer is common and women are interested in concurrent treatment. Currently, a multicenter study is planned, noteworthy is the very stringent inclusion criteria, as for endometrial cancer, concurrent treatment was offered to only 14.5% of women. In our opinion, sharp inclusion criteria are very important because the main goal of surgical treatment is to remove the tumor as radically as possible. In addition, a large proportion of patients will undergo radiotherapy treatment, the planning of which is disadvantaged by anatomical changes within the pelvis and may hinder or prevent proper follow-up treatment.

There is very little papers on concurrent treatment of urogynecological disorders and endometrial cancer and none of them are randomized control trials. Available papers focus on showing very low rate of performing concurrent surgery despite potential benefits and possibly no increased risk of postoperative adverse events. So far there are no data available on long term effectiveness of such treatment. A detailed list of qualified papers is presented in Table 8.

## 4. Summary

It is estimated that, in the general population, 25–30% of women suffer from pelvic floor disorders [28,36]. Based on the papers mentioned in the review we can conclude that those disorders are more frequent (50–60%) in women with endometrial cancer than in the general population [17,19]. However, no direct association of genitourinary dysfunction with oncologic disease has been found [48,49]. It is probable that high prevalence of the pelvic floor disorders in endometrial cancer patients is associated with certain risk factors which are common for both above mentioned health problems such as obesity, age over 50 years and certain comorbidities, for example, diabetes or hypertension. In the investigated group, the most frequent symptom is stress urinary incontinence and then as follows: urge urinary incontinence, pelvic organ prolapse and fecal incontinence [17,19]. Similar findings were described by Donovan et al. [49], who examined the prevalence of bladder and bowel symptoms in survivors of endometrial and cervical cancer in comparison with the prevalence in women without cancer. His study showed significantly higher rates of SUI, UUI, nocturia and urgency in cancer survivors.

The pelvic organs such as the reproductive organs, the gastrointestinal tract and the urinary tract are located close to each other, which implies the possibility of damage during surgical techniques. Among patients who have undergone surgical treatment, as much as 70–80% report pelvic symptoms [18,21], and urinary incontinence is the one with the biggest influence on quality of life [21]. Weakening of the fascial, muscular and nervous structures of the pelvis secondary to mechanical damage is the most common cause of pelvic organ prolapse [50]. During hysterectomy for oncological reasons, the vaginal stump is not fixed and thus the structures supporting the reproductive organ are not reconstructed, which increases the risk of subsequent disorders in pelvic floor statics. In a multicenter Polish study, it was found that 10% of patients reporting pelvic organ prolapse had a previous abdominal hysterectomy [11]. The literature also emphasizes the impact of intraoperative nerve damage that may lead to subsequent bladder dysfunction [10,12]. The prevalence of urinary tract symptoms does not depend on surgery technique and there is no statistically significant difference between groups treated with total abdominal hysterectomy (TAH), total laparoscopic hysterectomy (TLH), robotic surgery, neither does it matter if lymphadenectomy has been performed [18,20]. In our review we did not include the papers on sexual dysfunction and quality of life after endometrial cancer treatment, yet both of those aspects are connected to pelvic floor functioning. It is estimated that EC survivors have higher rates of dyspareunia and decreased sexual desire [51,52] than healthy controls. It turns out that the prevalence of sexual dysfunction does depend on surgery technique. Shisler, R et al. [52], in their review about patient-reported outcomes after endometrial cancer treatment, concluded that patients treated with laparoscopy compared to laparotomy had better QOL, sexual and vaginal functioning. Another study by R. Angioli et al. [53] compared quality of life of EC patients treated with or without systematic lymphadenectomy, and no statistically significant differences in QoL were found between groups. In conjunction with our results, it may be suspected that lifadanectomy does not affect the PFD and the QoL of patients with endometrial cancer.

The majority of papers qualified for the review focus on patients treated with radiotherapy as either part of the combined treatment or less commonly the only applied therapy. There were no significant differences in the incidence of pelvic floor functional disorders between the groups of patients undergoing surgery versus surgery followed by radiotherapy [22]. There is a lack of consistency in the incidence of toxicity, but the authors agree that early and late complications of EC radiotherapy more often affect the gastrointestinal tract than the urinary system [27,28,29,31]. Despite developing technology, it is still not possible to target radiation exclusively to the diseased organ—the bowel and bladder located in the immediate vicinity of the uterus also receive the radiation. Most complaints are mild and transient, are amenable to conservative therapy and rarely require re-hospitalizations. Most studies describe a decrease in the incidence and severity of toxicity over time [25,28,30]. The most common bowel toxicity symptom is diarrhea and fecal incontinence; the most common urinary toxicity is urge incontinence.

One of the possible problems affecting quality of life after VBT is vaginal atrophy [23]. There is a lower rate of complications, including pelvic floor dysfunction, after brachytherapy and IMRT teletherapy compared to conventional radiotherapy, which is reflected in the current standard of care [14]. In the systematic review about EC treatment outcomes Shisler et al. [52] show that there is no significant difference in QOL or sexual function between women treated with vaginal brachytherapy after surgery vs. surgery alone—this is another finding favoring choosing VBT over EBRT.

During the database search we did not find many papers on pelvic floor symptoms occurring after chemotherapy in patients with endometrial cancer, probably because chemotherapy is mainly used for advanced or disseminated cancer cases and mortality rates in this group are very high. Based on the papers qualified for the review, we can conclude there is no direct link between chemotherapy and urinary incontinence incidence. Authors describe increased prevalence of gastrointestinal symptoms after chemotherapy, nevertheless they seem to resolve quickly [9,16,33]

We noticed that treatment options for pelvic floor dysfunction following oncological treatment for endometrial cancer is very rarely the subject of research. The discussed studies describe promising results of oncological procedures with concomitant urogynecological surgery in patients diagnosed with urinary incontinence or pelvic organ prolapse prior to surgery [15,35]. We did not find any papers on management of urogynecologic disorders after completing the course of oncological treatment, this might be explained by rare urogynecological follow-up after endometrial cancer treatment, which leads to underdiagnosing pelvic floor disorders and lack of implementing adequate prophylaxis and treatment for EC survivors dealing with urinary incontinence or pelvic organ prolapse. In the review by Brennen R. et al. [54], the effects of pelvic floor muscle interventions on pelvic floor dysfunction after gynecological cancer treatment \were described. The authors concluded that pelvic floor muscle training supervised by a specialist was associated with improvement in quality of life. The results of literature review [55] demonstrate that PFMT is an effective treatment for UI in women, especially when it is supervised by a physiotherapist. There is not enough data on screening and following management of pelvic floor disorders in patients before and after endometrial cancer treatment, thus there is a need to carry out more research in the subject.

The material presented here is strong search terms, a systematic review of the available online literature and a valid methodological approach. There were very few papers that were entirely relevant to the topic, while those available were often conducted on small groups of patients and were mainly retrospective based on different questionnaires and in some cases without validation. The inclusion criteria we used result in a relatively small number of eligible articles. The articles reviewed often evaluate urinary dysfunction after combined treatment of several cancers together, most commonly cervical cancer and endometrial cancer [9,27,30,49]. The creation of such a heterogeneous study group does not fit into the inclusion criteria of our review, because in cervical cancer the surgical procedure is more radical and has a much greater impact on the statics of the reproductive organ than in endometrial cancer. At this stage, there is a lack of prospective studies in which, in addition to subjective assessment, clinical examination and evaluation of urinary incontinence were used.

## 5. Conclusions

Endometrial cancer coexists with such civilization diseases as diabetes, obesity and hypertension and the factors conducive to the disease include physical inactivity, low fertility, and an unhealthy high-calorie diet. The incidence of endometrial cancer is increasing rapidly and combined treatment has very good outcomes, as a result the population of endometrial cancer survivors is growing. Our systematic review suggests that pelvic floor disorders are common in endometrial cancer survivors and affect their quality of life. Clinicians should consider planning professional urogynecological evaluation at the moment of cancer diagnosis and screening for pelvic floor dysfunctions during treatment and follow-up. This can result in finding the patients that might benefit from conservative or surgical treatment for issues such as urinary incontinence or pelvic organ prolapse to improve their overall quality of life. Before cancer treatment, patients should be counselled about possible early and late pelvic floor symptoms and referred to physiotherapists for pelvic floor evaluation and education in the subject of PFMT. We believe that the evaluation and treatment of complications after endometrial cancer treatment will be an increasingly interesting and relevant research topic and there will be more prospective studies helping clinicians with developing optimal care standards.

## Figures and Tables

**Table 1:** ROBINS analysis.

Author	Type of Study	Bias Due to Confounding	Bias in Selection of Participants	Bias Due to Missing Data	Bias in Measurement of Outcomes	Bias in Selection of the Result	Overall
Emirdar, 2016	Prospective cohort	Serious	Low	Low	Moderate	Low	Moderate
Bretschneider, 2018	Retrospective cohort	Serious	Serious	Moderate	Moderate	Serious	Serious
Bahng, 2012	Retrospective	Serious	Moderate	Moderate	Moderate	Serious	Serious
Bochenska, 2018	Retrospective	Serious	Moderate	Moderate	Moderate	Serious	Serious
Bretschneider, 2016	Cross-sectional	Serious	Moderate	Moderate	Moderate	Serious	Serious
Kaufmann, 2016	Comparative study	Serious	Serious	Low	Moderate	Serious	Serious
Kuku, 2013	Retrospective	Serious	Moderate	Moderate	Moderate	Serious	Serious
Lipetskaia, 2019	Retrospective	Serious	Moderate	Moderate	Moderate	Serious	Serious
Samper-Ternert, 2011	Retrospective	Serious	Moderate	Moderate	Moderate	Serious	Serious
Nosti, 2012	Cross-sectional	Moderate	Moderate	Low	Moderate	Moderate	Moderate
Opławski, 2015	Cross-sectional	Serious	Serious	Moderate	Moderate	Serious	Serious
Robison, 2016	Prospective cohort	Serious	Serious	Moderate	No info	No info	Critical
Roszak, 2012	Prospective	Serious	Low	Moderate	Moderate	Moderate	Moderate
Segal, 2018	Retrospective	Serious	Moderate	Moderate	Moderate	Serious	Serious
Soisson, 2017	Prospective cohort	Moderate	Moderate	Moderate	Serious	Serious	Serious
Thomas, 2013	Cross-sectional	Moderate	Moderate	Moderate	Moderate	Moderate	Moderate
Vandecasteele, 2011	Prospective cohort	Moderate	Moderate	Moderate	Moderate	Serious	Serious

**Table 2:** Characteristics of publications qualified for the review.

Author	Surgery	RTH	ChTH	UI	FI	POP	RTH Toxicity
Bretschneider CE et al., 2016 [19]				+	+		
Thomas SG et al. 2012 [17]				+	+	+	
Lipetskaia et al., 2019 [18]	+			+			
Higgs et al., 2017 [20]	+			+		+	
Nosti et al., 2012 [21]	+			+		+	
Segal, S. et al., 2017 [22]	+	+		+	+	+	
de Boer et al., 2015 [14]	+	+		+	+		
Opławski M et al., 2015 [23]	+	+		+			
Bahng AY et al., 2012 [24]	+	+					+
Nout et al., 2011 [13]	+	+		+			
Kauffmann G et al., 2017 [25]		+					+
Soisson, S et al., 2018 [16]	+	+	+				
Emirdar et al., 2016 [26]	+	+		+			
Kuku et al., 2013 [27]	+	+					+
Vandecasteele, K et al., 2012 [28]	+	+					+
Barillot I et al., 2014 [29]	+	+					+
Roszak A et al., 2012 [30]	+	+					+
Samper-Ternent, R et al., 2011[31]	+	+					+
Onsrud M et al., 2013 [32]	+	+					+
de Boer et al., 2016 [33]	+	+	+	+			
de Boer et al., 2019 [9]	+	+	+	+			
Robison et al., 2016 [34]	+			+			
Bretschneider C et al., 2018 [15]	+			+		+	
Bochenska et al., 2018 [35]	+				+		

US = urinary symptoms, BS = bowel symptoms, RTH = radiotherapy, ChTH = chemotherapy, UI = urinary incontinence, FI = fecal incontinence, POP = pelvic organ prolapse, PF = pelvic floor, SNS = sacral nerve stimulation, QOL = Quality of Life.

**Table 3:** Pelvic floor dysfunction in women with EC before treatment.

Author	Objective	Participants	Assessment	Pelvic Floor Dysfunction in EC, *n* (%)					Summary
				UI	SUI	UUI	FI	POP	
Bretschneider et al., 2016 [19]	To assess prevalence of PFD in women with suspected GM.	152 women with GM aged 58.1 ± 13.3 including 94 women with EC. BMI: 33.6 ± 8.8	RSC, ICIQ-FLUTS	35 (37)	27 (29)	23 (24)	3 (3)	N/I	Pelvic floor disorders are common in women with suspected gynecological malignancies.
Thomas et al., 2012 [17]	To assess prevalence of PFD in women with suspected GM.	549 women aged 59.6±0.9: 347 (63.2%) women with GM, including 189 (49.9%) women with EC. 202 (36.8%) with benign gynecological condition BMI: 32.2 ± 0.5	One page questionnaire on pelvic floor dysfunction based on PFDI	102 (55)	67 (36)	27 (14.5)	N/I	13 (7)	The prevalence of UI at baseline was similar among patients with all types of gynecologic cancer. No significant difference was observed in prevalence or severity of UI and POP between women with benign vs. malignant gynecology disease.

UI = urinary incontinence, SUI = stress urinary incontinence, UUI = urgency urinary incontinence, FI = fecal incontinence, POP = pelvic organ prolapse, PFD = pelvic floor disorders, GM = gynecological malignancy, BMI = body mass index, LUTD = lower urinary tract disfunction, RSC= Rotterdam Symptom Checklist, ICIQ-FLUTS = International Consultation on Incontinence Questionnaire-Female Lower Urinary Tract Symptoms, N/I = no information, PVR = post voiding residue, USG TV = transvaginal ultrasonography, PFDI = pelvic floor distress inventory.

**Table 4:** Pelvic floor dysfunction in EC patients after surgical treatment.

Author	Objective	Participants	Assessment	Intervention	PFD Preop.	PFD Postop.	Outcomes
Lipetskaia et al., 2019 [18]	To assess the long term effect of lymph node dissection on LUTS in patients treated for EC.	74 women with EC—FIGO stage I Control group: 37 women aged 58 ± 11 Study group: 37 women aged 60 ± 11	UDI-6, IIQ-7 after 13 ± 8 months	Robotic-assisted TLH and BSO; Control group: w/o additional intervention Study group: with lymph node dissection	Total UI: 28%	Total UI: 74.3% Control group: IIQ-7 14.9+/−23 UDI-6 30.0+/−25.3 Study group: IIQ-7 10.5+/−22.9(*p* = 0.419) UDI-6 20.7+/−22.9(*p* = 0.104) The odds ratio for developing new-onset UI—2.4 with 95% CI 0.62–9.5 (*p* 1⁄4 0.18).	There was a significant increase in the incidence of urinary incontinence after robotic-assisted TLH and BSO. No statistically significant increase in the incidence of UI in the study group. No statistically significant differences in IIQ-7 and UDI-6 score depending on lymph node dissection.
Higgs et al., 2017 [20]	To assess PFD after treatment for early stage EC in patients who underwent TAH or TLH.	381 women with EC FIGO stage IA 195 women aged 62.6 ± 10.9 underwent TAH 186 women aged 63.0 ±9.5 underwent TLH	PFDI preop. and 6, 18, 30, 42, 54 months postop.	Patients were randomly allocated to TAH or TLH	Moderate to severe symptoms TAH: US: 6% POP: 5% CRAS: 4% TLH: US: 11% POP: 8% CRAS: 7%	Moderate to severe symptoms after 6 months TAH: US: 5% POP: 2% CRAS: 5% TLH: US: 6% POP: 3% CRAS: 4%	No statistically significant increase in the incidence of PFD in terms of urinary, bowel and prolapse symptoms in both groups after 6, 18, 30, 42 and 54 months postop. After 6 months all patients showed improvement from baseline in the POP and urinary stress domain.
Nosti et al., 2012 [21]	To assess prevalence of PFD in postoperative patients with EC and the impact of these issues on QoL.	25 women aged 62 (±12) with EC FIGO stage IB. BMI 32(19–46)	PFDI-20 and PFIQ-7 after 19 (6–42) months	TAH with BSO ± lymph node sampling	N/I	Any symptoms: US: 76% POP: 44% CRAS: 68% Moderate to severe symptoms: US: 24% POP: 12% CRAS: 12%	Pelvic symptoms were reported by 84% patients (mild: 76%; severe: 8%) Quality of life issues associated with PFS: 44% patients. Pelvic symptoms were reported at a much higher rate than seen in the general public.

LUTS = lower urinary tract symptoms, EC = endometrial cancer, FIGO = International Federation of Gynaecology and Obstetrics, UDI-6 = Urinary Distress Inventory, IIQ-7 = Incontinence Impact Questionnaire, TLH = total laparoscopic hysterectomy, BSO = bilateral salpingo-oophorectomy, PFD = pelvic floor dysfunction, TAH = total abdominal hysterectomy, PFDI = pelvic floor distress inventory, QoL = quality of life, PFIQ-7 = Pelvic Floor Impact Questionnaire, US = urinary symptoms, preop. = preoperatively, postop. = postoperatively, CRAS = colorectal anal symptoms, N/I = no information.

**Table 5:** Pelvic floor dysfunction in EC patients after radiotherapy.

Author	Objective	Participants	Assessment	Intervention	Follow-Up Time	Outcomes
Segal, S. et al., 2017 [22]	To assess the prevalence of UI, FI, POP and sexual dysfunction in patients who underwent RTH for EC.	159 women with EC FIGO stage I (radiation data available for 149 patients). Control group: 87 women Study group: 62 women	ISI, QUID, FISI, PFDI-20 question No 3, PISQ-12	Surgery: hysterectomy via laparotomy, (85) minimally invasive techniques (60), or no surgery (4) Control group: no RTH Study group: 28 VBT, 34 EBRT	After 8–10 years from diagnosis	Symptoms no RTH vs. RTH UI 57.5% vs., 48.5% *p* = 0.47 FI 48.3% vs. 45.2% *p* = 0.66 POP 3.4% vs. 6.5% *p* = 0.33 Sexual function score (median) 32 vs. 21 *p* = 0.03
de Boer et al., 2015 [14]	To assess the long-term outcome, HRQL, urinary and bowel symptoms and sexual functioning of patients with stage I high-intermediate risk EC treated with EBRT or VBT	427 women with FIGO stage I high-intermediate risk EC	EORTC-QLQ-C30	214 women aged 69.3(51–89) received EBRT 213 women aged 69.8(46–85) received VBT	After 7 and 10 years	Symptoms (severe to moderate): UU EBRT 39.3%, VBT 25.5% *p* = 0.05 UI EBRT 11.9% VBT 8.7% *p* = 0.89 FL EBRT 10.6%, VBT 1.8% *p* = 0.0 Symptoms (mild, moderate or severe): UU EBRT 67,9% VBT 61,3% UI EBRT 42,9% VBT 45,2% FL EBRT 24,7% VBT 15%
Opławski M et al., 2015 [23]	To evaluate urinary tract function and QoL in EC patients after combined treatment.	46 women: 23 EC stage IA patients (G1-G2) 23 non-oncological patients	Medical history, gynecologic and urodynamic evaluation, STAI, BDI	Study group: Radical hysterectomy and VBT Control Group: Non-oncological hysterectomy with uterine appendage removal	6–12 months after surgical treatment	Significant difference between the two groups in terms of urogynecological outcomes (*p* = 0.0193). Study group (%): SUI = 26 MUI = 39 OAB = 13 Control group (%): SUI = 4 MUI = 22 OAB = 9
Bahng AY et al., 2012 [24]	To evaluate prognostic factors and occurrence rates of radiation-induced vaginal mucosal toxicity in patients who have received BRT for EC.	100 EC patients aged 41–84	CTCAE v. 4.02.	Total hysterectomy + BSO with or without lymph node dissection and adjuvant VBT Study group: vaginal dilator use Control group: no dilator	24 months (4 months to 14 years)	The incidence of Grade 1 or asymptomatic vaginal toxicity was 33% and Grade 2–3 or symptomatic vaginal toxicity was 14% Use of vaginal dilator at least 1×/week was associated with decreased vaginal mucosal toxicity
Nout et al., 2011 [13]	To assess the long-term outcome and HRQL of patients with EC treated with or without pelvic RTH.	714 patients with stage I EC 113 women after EBRT returned questionnaire 133 women with NAT returned questionnaire	EORTC, SF-36	TLH with BSO Study group: EBRT Control group: NAT	After 13.3 years (2.8–18.5)	Women treated with EBRT reported lower scores on all scales of the SF-36 SF-36 scores: UU EBRT 46, NAT 32 *p* = 0.001 UI EBRT 30, NAT 16 *p* < 0.001 Need to remain close to toilet EBRT 26 NAT 10 *p* < 0.001 Fecal urgency EBRT 44 NAT 25 *p* < 0.001 FI EBRT 19 NAT 8 *p* = 0.002 Diarrhoea EBRT 25 NAT 10 *p* < 0.001
Kauffmann G at al., 2017 [25]	To evaluate acute and late toxicity after triple-tandem high—dose VBT for medically inoperable EC.	6 women with medically inoperable EC stage I aged 57 (53–70). BMI: 49.8 (44.8–76.5)	History, physical examination, CTCAE v. 4	Triple-tandem high - dose VBT with (3) or without (3) preceding EBRT	Median follow-up of 6.5 months	EBRT + VBT: Acute GI toxicity 3/3 patients (grade 1–2) Acute GU toxicity 3/3 patients (grade 1–2) HDR VBT: Acute GI toxicity 2/6 patients (grade 1–2) Acute GU toxicity 2/6 patients (grade 1)
Soisson, S et al., 2018 [16]	To evaluate the urinary system and genital system disorders among EC survivors.	2648 EC survivors and 10,503 individuals from the general population	ICD-9 diagnosis codes (genitourinary/urinary system, genital organs), ambulatory inpatient and surgery records	Study groups: S/S + ChTH/S + RTH/S + ChRTH Control group: general population	After 1–5 and 5–10 years	Urinary system disorders—HR: Surgery Surgery+ RTH (1–5 years): 1.46 Surgery+ RTH (5–10 years): 1.24 Genital system disorders: Surgery Surgery+ RTH (1–5 years): 1.26 Surgery+ RTH (5–10 years): 1.09
Emirdar et al., 2016 [26]	To assess the short-term effects of adjuvant or primary curative RTH on the urinary system in women with gynecologic cancer.	55 women: Group 1: 10 women with early stage cervical cancer aged 46.6 ± 8.6 Group 2: 36 women with EC aged 59.5 ± 5.2 Group 3: 9 women with IIB or advanced cervical cancer aged 46.6 ± 10.2	Urodynamic examination	Group 2: TAH + BSO, pelvic + para-aortic lymph node dissection and omentectomy + adjuvant RTH	Before and 6 months after treatment	Group 2 (EC): Incontinence: preT 27.8%, postT 38.9%, *p* = 0.046 Positive UM: preT 33.3% postT 22.2% *p* = 0.157 FUUV: preT 195 ± 80.1 postT 186.9 ± 89.1 *p* = 0.649 NUUV: PreT 351.2 ± 119.0 PostT 301.8 ± 101.7 *p* = 0.037 SUUV PreT 485.3 ± 145.3 PostT 393.8 ± 122.8 *p* = 0.000 Bladder capacity (ml) PreT 600.2 ± 124.8 PostT 490.0 ± 92.6 *p* = 0.000 Residual urine (ml) preT 4.0 ± 1.3 postT 4.1 ± 1.0 *p* = 0.914 MVP preT 129.3 ± 40.1 postT 130.2 ± 56.9 *p* = 0.793 MDP preT 69.4 ± 23.8 postT 77.7 ± 41.6 *p* = 0.338

UI = urinary incontinence; FI = fecal incontinence; POP = pelvic organ prolapse; RTH = radiotherapy, EC = endometrial cancer; HRQL = health-related quality of life; EBRT = external beam radiation therapy; VBT = vaginal brachytherapy; FIGO = International Federation of Gynaecology and Obstetrics; EORTC-QLQ-C30 = European Organisation for Research and Treatment of Cancer quality of life questionnaire ; UU = urinary urgency SUI = stress urinary incontinence, MUI = mixed urinary incontinence; STAI = State-Trait Anxiety Inventory, BDI = Beck Depression Inventory, CTCAE = Common Toxicity Criteria for Adverse Events, NF = nocturnal frequency; PORTEC-1 = Post-Operative Radiation Therapy in Endometrial Carcinoma 1; TAH = total abdominal hysterectomy BSO = bilateral salpingo-oophorectomy; preT = pretreatment; postT = post treatment; CHTH = chemotherapy; QoL = Quality of Life; PFS = pelvic floor symptoms; PFDI = pelvic floor distress inventory; PFIQ-7 = Pelvic Floor Impact Questionnaire; GI = gastrointestinal, GU = genitourinary, FL = fecal leakage; SF-36 = Short Form 36-Item; ISI = Incontinence Severity Index questionnaire, QUID = Questionnaire for Urinary Incontinence Diagnosis, FISI = Fecal Incontinence Severity Index, PISQ-12 = Pelvic Organ Prolapse/Urinary Incontinence Sexual questionnaire.

**Table 6:** Acute and late bladder and bowel toxicities among EC patients treated with radiotherapy.

Author	Study Objective	Participants	Follow Up	Assessment	Intervention (EC Patients)	Acute GI Toxicity	Acute Bladder Toxicity	Late GI Toxicity	Late Bladder Toxicity
Kuku et al., 2013 [27]	To describe the symptoms of radiotherapy-induced bowel injury.	541 women: 219 women with CC aged 52 (27–81) 322 women with EC aged 63 (40–80). Selected for analysis: 77 CC patients, 73 EC patients	3 months up to 10 years	Clinical examination, routine screening for bowel and bladder toxicity	S: TAH/TLH +BSO with peritoneal washings + ChTH (36% of patients) RTH: EBRT (100% of patients) Dose: 45 Gy in 25 fractions + 12 Gy in 2 fractions to the vaginal vault	N/I	N/I	73 EC patients (23%) reported bowel toxicity: defecation urgency 8.7%, frequency < 4/day 65.3%, diarrhea 48%, pain 45.3%, bloating 30.7%, FI 21.3%	N/I
Vandecasteele, K et al., 2012 [28]	To evaluate acute and late toxicity after postoperative IMAT for EC.	65 women: 41 women with EC aged 67 (50 to 83) 24 women with CC aged 49 (35–71)	Weekly during IMAT + after IMAT: 1,3 months, then every 3–6 months (years 1–5)	RTOG scoring system, the scale of GI urgency and incontinence in-house developed scales for rectal blood loss and UI, Radiation-Induced Lower Intestine scoring scale	S: TAH + BSO with or w/o pelvic lymphadenectomy OR resection of the local recurrence RTH: IMAT followed by VBT or an external boost if VBT was not feasible Para-aortic irradiation if PALN were affected Dose: 46 Gy in 23 fractions + 11–21 Gy	93% Most of the toxicity was grade 1 Frequency 85%, abdominal cramps 56%, urgency 34%, nausea 29%, incontinence 7%	63% Most of the toxicity was grade 1. Nycturia 46%, urge 41%, pollakiuria 34%, dysuria 32%, Incontinence 14%	36% Most of the toxicity was grade 1, no grade 3 toxicity Frequency 20%, cramps 8%, urgency 4%, Mucus loss 4%, Abdominal discomfort 4%	36% All grade 1–2 Incontinence 20%, pollakiuria 12%, urge 8%, dysuria 4%, nycturia 4%
Barillot I et al., 2014 [29]	To evaluate acute toxicity (grade 2 or higher) after IMRT for EC.	46 women with EC (stage I-II) aged 65.5 (57–75)	During IMRT + within 90 days	CTCAE-3.0	S: TAH/TLH +BSO. Pelvic lymphadenectomy in 96% of patients RTH: IMRT, 36 patients received additional HDR VBT Dose: 45.5 Gy (median)	85%. Grade 2: <30%. Grade 3: 0%	39.5%. Grade 2: <20% Grade 3: 0%	N/I	N/I
Roszak A et al., 2012 [30]	To evaluate acute and late toxicity after radiotherapy for gynecological cancer.	263 patients with CC (*n* = 128) and EC (*n* = 135) treated with definitive (CC) or adjuvant RTH (CC and EC)	Weekly during treatment + 2 years	EORTC/RTOG toxicity scale	S: not specified RTH: EBRT + HDR VBT. Dose: 43.4 Gy + 18 Gy in 63 fractions	26.5% Most was grade 0 (73.3%) and grade 1–2 (17.8%)	18.5% Most was grade 0 (81,5%) and grade 1–2 (17.0%)	7.4% Most of the toxicity was grade 0 (92.6%). There were no patients with toxicity of grade 3 or above	1.5% Most of the toxicity was grade 0 (98.5%). There were no patients with toxicity of grade 3 or above
Samper-Ternent, R et al., 2011 [31]	To evaluate acute and late toxicity after radiotherapy for EC.	8797 women with EC	60 months	Database search for any gastrointestinal or bladder diagnosis based on ICD-9-CM codes	S: 87% had surgery, type not specified Study group: EBRT or radioactive implants or BOTH Control group: w/o radiation. Dose: not specified	Radiation 21.9% No radiation 17.5% (*p* < 0.0001). Any grade Inflammation 12.5%, hemorrhage 4.9%, Obstrucion 3.9%	Radiation 14.3% No radiation 15.5% (*p* = 0.1398) Any grade hemorrhage 4.7%, incontinence 4.6%	Radiation 60.8% No Radiation 53.1% (*p* < 0.0001) Any grade inflammation 42.2%, hemorrhage 31.5%, obstruction 12.4%	Radiation 35.8% No Radiation 31.9% (*p* = 0.0004) Any grade incontinence 17.3%, hemorrhage 17.3%, cystitis 10%
Onsrud M et al., 2013 [32]	To evaluate long-term effects of EBRT treatment for early-stage EC.	568 EC patients, 280 with adjuvant VBT, 288 with adjuvant VBT + EBRT	20+ years	French-Italian Glossary (toxicity)	S: TAH + BSO Study group: VBT + EBRT Control group: VBT Dose: 60 Gy (VBT), 40 Gy (EBRT) in 20 fractions	Study group: 27.4% grade 2 toxicity or higher, 2.9% grade 3–4 toxicity. Control group: 4.5% grade 2 toxicity, no reports of grade 3 or 4 toxicity Median survival time: study group: 20.48 years, control group 20.5 years (*p* = 0.186)			

CC = cervical cancer, EC = endometrial cancer, TAH = total abdominal hysterectomy, S = surgery, TLH = total laparoscopic hysterectomy, BSO = bilateral salpingo-oophorectomy, ChTH = chemotherapy, RTH = radiotherapy, EBRT = external beam radiotherapy, Gy = gray, IMRT = Intensity Modulated Radiotherapy, CTCAE-3.0 = Common Terminology Criteria for Adverse Events v3.0, HDR VBT = high dose rate vaginal brachytherapy, EORTC = European Organisation for Research and Treatment of Cancer, RTOG = Radiation Therapy Oncology Group, ICD-9-CM = International Statistical Classification of Diseases and Related Health Problems 9-CM.

**Table 7:** Pelvic floor disorders in patients with EC treated with RTH and CHRTH.

Author	Objective	Participants	Assessment	Intervention	Outcomes
de Boer et al., 2016 [33]	To assess the benefit of adjuvant ChRTH compared with RTH alone for women with high-risk EC. To assess 2-year toxicity and QoL.	686 women with EC underwent TAH/TLH with BSO and next were randomly allocated (1:1) to receive either CHRTH or RTH alone. RTH group: 333 women aged 61.9 (55.9–68.1) CHRTH group: 327 women aged 62.5 (56.5–68.0)	Common Terminology Criteria for Adverse Events version 3.0 and EORTC QLQ-C30, CX24, OV28 after RTH and at 6, 12, 24, 36 and 60 months after randomization	RTH group underwent pelvic RTH 48.6 Gy in 1.8 Gy fractions, five times a week for 5.5 weeks. CHRTH group additionally received 2 cycles of cisplatin 50 mg/m^2^ in the first and fourth week of RTH, followed by 4 cycles of carboplatin area under the curve (AUC) 5 and paclitaxel 175 mg/m^2^ at 21-day intervals (and a 28-day interval between the second concurrent and first adjuvant cycle).	CHRTH for high-risk EC caused significantly higher incidence of severe adverse gastrointestinal events and reduced health-related quality of life during treatment compared with RTH alone, but with rapid recovery. After 6 and 12 months, there was no significant difference between groups in bowel and urinary symptoms.
de Boer et al., 2019 [20]	To compare five-year survival, recurrence and adverse events in women with high-risk EC treated with ChRTH compared with RTH alone.	686 women with EC underwent TAH/TLH with BSO and next were randomly allocated (1:1) to receive either CHRTH or RTH alone. RTH group: 330 women aged 62.0 (55.8–68.2) CHRTH group: 330 women aged 62.4 (56.5–67.9)	Common Terminology Criteria for Adverse Events version 3.0 5 years after randomization	RTH group underwent pelvic RTH 48.6 Gy in 1.8 Gy fractions, five times a week. CHRTH group additionally received 2 cycles of cisplatin 50 mg/m^2^ in the first and fourth week of RTH, followed by 4 cycles of carboplatin area under the curve (AUC) 5 and paclitaxel 175 mg/m^2^ at 21-day intervals (and a 28-day interval between the second concurrent and first adjuvant cycle).	CHRTH for high-risk endometrial cancer did not improve five-year overall survival, but increase failure-free survival. No significant differences between treatment groups in genitourinary and gastrointestinal adverse events at 60 months.
Soisson at al., 2018 [16]	To assess the urinary system and genital system disorders among EC survivors.	2648 EC survivors and 10,503 individuals from the general population.	ICD-9 diagnosis codes (genitourinary/urinary system, genital organs), ambulatory inpatient and surgery records	Surgery / surgery + ChTH/surgery + RTH/surgery + ChTH + RTH	EC survivors have higher incidence of urinary system and genital system disorders than general population. Patients treated with surgery in combination with RTH and/or ChTH were at higher risk for both urinary system and genital organ disorders compared to those treated with surgery alone. Stage at diagnosis was not associated with risk for genital organ disorders. Higher EC grade was associated with higher risk for urinary system disorders. Higher BMI was not strongly associated with urinary system or genital organ disorders

ChRTH = chemo-radiotherapy; RTH = radiotherapy; EC = endometrial cancer; EORTC QLQ-C30 = European Organization for Research and Treatment of Cancer; QLQ-C30 = Quality of Life Questionnaire; QLQ-CX24 = Cervical Cancer-Specific Quality of Life Questionnaire; QLQ-OV-28 = Ovarian Cancer-Specific Quality of Life Questionnaire; ICD-9 = International Classification of Diseases; BMI = body mass index, UI = urinary incontinence, UF = urinary frequency; USD = urinary system disorders; GOD = genital organ disorders.

**Table 8:** Treatment of pelvic floor disorders after EC treatment.

Author	Objective	Participants	Assessment	Intervention	Objective	Outcomes
Bochenska et al., 2018 [35]	To evaluate the rate of POPUI procedures in women undergoing surgery for a gynecologic malignancy.	23,501 women with EC (*n* = 14,711), ovarian cancer (*n* = 5961), cervical cancer (*n* = 1922)	Primary surgery with or w/o concurrent procedure for PFD (SUI, POP, both)	ACS-NSQIP database analysis	*n*/i	434 (2.9%) EC patients underwent concomitant POPUI procedures. In the whole group 76.9% of surgeries were performed for repair of POP, 23.1% were performed for SUI. No statistically significant difference in serious adverse events 30 days post operatively between groups.
Bretschneider C et al., 2018 [15]	To evaluate the outcomes after concurrent surgeries for gynecology cancer and PFD—retrospective study.	25,138 cases of gynecological cancer	Primary surgery with or w/o concurrent procedure for PFD (SUI, POP, both)	*ACS NSQIP* database analysis ICD-9 codes	30 days	2.3% (589) patients underwent concurrent procedure for PFD, most commonly it was POP procedure. There was no statistically significant difference in adverse effects incidence between groups. The group that might benefit the most from concurrent PFD surgery is EC patients rather than CC or OC patients.
Robison at al., 2016 [34]	To assess whether EC patients could be screened for SUI before treatment and then referred for concurrent treatment of EC and SUI.	59 women aged 62.1 (37–85) with early stage (IA-IIIC) EC	anti-incontinence concurrent surgery OR non-surgical SUI treatment OR SUI observation	author’s questionnaire, urogynecological examination	32 days (14–60)	Baseline incidence of SUI: 39% (20) 80% (16) of patients screened positive for SUI wanted urogynecological consult before the surgery. 8 (53%) patients with EC and SUI wanted to undergo anti-incontinence concurrent surgery.

POPUI = pelvic organ prolapse urinary incontinence, EC = endometrial cancer, PFD = pelvic floor disease, SUI = stress urinary incontinence, POP = pelvic organ prolapse, ACS NSQIP = American College Of Surgeons National Surgical Quality Improvement Program, CC = cervix cancer, OC = ovarian cancer.
